# Three Cases of Vaginal Hysterectomy for Malignant Disease

**Published:** 1897-03

**Authors:** W. H. C. Newnham

**Affiliations:** Assistant Physician-Accoucheur, Bristol General Hospital


### THREE CASES OF VAGINAL HYSTERECTOMY FOR MALIGNANT DISEASE.

BY

W. H. C. Newnham, M.A., M.B. Cantab., M.R.C.S. Eng.,

*Assistant Physician-Accoucheur, Bristol General Hospital.*

Unfortunately in the majority of cases of malignant disease of the uterus which are met with, the growth is so far advanced that it is quite hopeless to attempt anything in the way of a radical operation, and one has to be content simply with palliative measures. Under these circumstances it occurred to me that it might be interesting to record the three following cases which have been successful, and up to the present time

[1] Read at the January meeting of the Bristol Medico-Chirurgical Society. The discussion on the paper is reported under "Meetings of Societies."

are all doing well. If the operation of vaginal hysterectomy is as successful as is claimed by Jessett and others, who have operated now upon a very large number of patients, then it becomes the duty of every medical practitioner to be on the look-out for any of the early signs of malignant disease of the uterus, in order that the operation may be undertaken before the disease has spread into the broad ligaments.

I will give short extracts of each case; and I am greatly indebted to my two clerks, Mr. Foss and Mr. Moorshead, for their very interesting and voluminous notes, which are too long to be reproduced in full:

CASE 1.—S. B., aged 29. No history of cancer in the family, except one distant relative on the mother's side. Was married at 16 years of age, and at the age of 21 had had five children. Between the ages of 21 and 29 she had two miscarriages. She was admitted on May 18th, 1896. The history of any uterine trouble dated from the previous September; from this month she gives notes of pain, various irregular hemorrhages, getting thinner, offensive discharge between the hemorrhages, and every week she was getting more markedly anæmic. On admission the hemorrhage was so great that she was plugged with gauze.

After consultation with my colleagues, I performed the operation of complete hysterectomy on June 9th. I used Jessett's needle and ligatured the broad ligaments on each side with silk, not using any clamp at all.

Three days after the operation she had a considerable amount of peritonitis and appeared to be in a very critical condition; but under appropriate treatment this subsided, and she was discharged well on July 25th.

CASE 2.—F. S., aged 38. Admitted to the General Hospital on June 9th, 1896. No history of malignant disease in the family. Married twice, first at the age of 17, and has had ten children in fourteen years. She was a widow for seven years. She also had three miscarriages. She has complained of pain for the twelve months before admission, and seven months ago she was confined with twins. For the last month only has she complained of irregular hemorrhage. During the last month the bleeding has been so great as to cause her to faint, and she has also kept her bed during that time. On admission she was markedly anæmic from the great loss. She was found to have an extensive malignant growth of the cervix, but it had not involved the broad ligaments.

On June 18th, after consultation, I removed the whole of the uterus *per vaginam*, again using Jessett's needle, and ligaturing each broad ligament with strong silk. The uterus was studded at the fundus with small growths.

This patient also on the third and fourth days after the operation had a considerable amount of peritonitis; but it subsided, and she was discharged well on August 7th.

CASE 3.—E. A., aged 39, was sent to me for operation from Devonshire. She was admitted on September 18th, 1896. No history of cancer in the family. Married at the age of 21, and has had ten children in eighteen years. Six years previously, when pregnant with her eighth child, she noticed that she had irregular hemorrhage up to 4½ months. Also while pregnant with her last child (which was born five months ago) she had a hemorrhage every month during the last six months of pregnancy. She had very considerable hemorrhage at the birth of the child, and has hardly been clear for one day since; she has also noticed that the discharge is most offensive. She was found to have malignant growth of the cervix extending on to the posterior vaginal wall for some distance.

On September 24th, after consultation, I removed the whole uterus *per vaginam*, using Jessett's needle, and ligaturing each broad ligament. After removal it was noticed in this case that the coils of intestine were in full view, whereas in the first two I had not seen them.

This patient had no signs of peritonitis after the operation, but made an uninterrupted recovery; she had no rise of temperature, and was able to take solid food more quickly than either of the preceding patients. As my clerk properly says, she was most cheerful and tractable, recognising that quietness was an essential part of the treatment. She was discharged well on November 19th.

What are the points for discussion that may be raised on the three cases just read? I think there are three very interesting ones.

(I.) The operation itself. Is it justifiable? That it can be done successfully there can be no doubt, but will the disease recur? Time alone can answer this question. There is no great difficulty about the operation, but at the same time I do not envy the beginner his first vaginal hysterectomy.

(II.) Is it better in these cases of disease of the cervix only to remove the whole uterus, or to do supra-vaginal amputation? There are many and various opinions on this point; but I have seen so many cases of recurrence after the minor operation, that I am at present strongly in favour of the major one.

(III.) Is there any reason to account for such an early development of malignant disease? Here we have three young women of 28, 38, and 39 respectively, all married very early in life: the first has five children between 16 and 21 years of age; the second is married at 17 and has ten children in a comparatively short time; and the third, although not married quite so young (21), still has ten children very quickly. Is it possible that very early and very frequent child-bearing may have a tendency to produce development of malignant disease in the uterus?

